# Automatic Identification of Information Quality Metrics in Health News Stories

**DOI:** 10.3389/fpubh.2020.515347

**Published:** 2020-12-18

**Authors:** Majed Al-Jefri, Roger Evans, Joon Lee, Pietro Ghezzi

**Affiliations:** ^1^Department of Medicine, Cumming School of Medicine, University of Calgary, Calgary, AB, Canada; ^2^Data Intelligence for Health Lab, Cumming School of Medicine, University of Calgary, Calgary, AB, Canada; ^3^School of Computing, Engineering and Mathematics, University of Brighton, Brighton, United Kingdom; ^4^Department of Community Health Sciences, Cumming School of Medicine, University of Calgary, Calgary, AB, Canada; ^5^Department of Cardiac Sciences, Cumming School of Medicine, University of Calgary, Calgary, AB, Canada; ^6^Brighton & Sussex Medical School, Falmer, Brighton, United Kingdom

**Keywords:** online health information, machine learning, health information quality assessment, text classification, natural language processing

## Abstract

**Objective:** Many online and printed media publish health news of questionable trustworthiness and it may be difficult for laypersons to determine the information quality of such articles. The purpose of this work was to propose a methodology for the automatic assessment of the quality of health-related news stories using natural language processing and machine learning.

**Materials and Methods:** We used a database from the website HealthNewsReview.org that aims to improve the public dialogue about health care. HealthNewsReview.org developed a set of criteria to critically analyze health care interventions' claims. In this work, we attempt to automate the evaluation process by identifying the indicators of those criteria using natural language processing-based machine learning on a corpus of more than 1,300 news stories. We explored features ranging from simple n-grams to more advanced linguistic features and optimized the feature selection for each task. Additionally, we experimented with the use of pre-trained natural language model BERT.

**Results:** For some criteria, such as mention of costs, benefits, harms, and “disease-mongering,” the evaluation results were promising with an F_1_ measure reaching 81.94%, while for others the results were less satisfactory due to the dataset size, the need of external knowledge, or the subjectivity in the evaluation process.

**Conclusion:** These used criteria are more challenging than those addressed by previous work, and our aim was to investigate how much more difficult the machine learning task was, and how and why it varied between criteria. For some criteria, the obtained results were promising; however, automated evaluation of the other criteria may not yet replace the manual evaluation process where human experts interpret text senses and make use of external knowledge in their assessment.

## 1. Introduction

Health information quality (HIQ) is a major public health issue because low-quality information can expose health professionals, patients, and the public to the risk of forming potentially harmful beliefs (1, 2). It has been found that struggling to get timely appointments as well as financial barriers to health care access render more people to seek health information online (3). Recently, concerns have focused on online information, as the inherently unregulated nature of the Internet allows anyone to post incorrect information. This has led to the development of several instruments designed for assessing HIQ of websites. These include, among others, the JAMA score (4), the DISCERN criteria (5), and the HON certification (6). These instruments, originally aimed at providing the public with tools to identify trustworthy websites, have been widely used in academic research on online HIQ.

News outlets, in particular, are disseminating the same information through different media (TV, radio, magazines, newspapers, and their respective websites). Such news stories, particularly those reporting on research about new treatments, are often solely based on press releases from biotech or pharmaceutical companies or from the communication offices of universities that want to promote their research. Although research on the quality of health news stories in the press is not as widely studied as HIQ of websites, studies have shown that these can be biased, exaggerated and lacking basic fact-checking (7, 8). For the purpose of this study we used health information available online because that is present in the healthnewsreview database that we utilized. It should be noted that this includes both online-only sources and print media that are also present online. Of note, the database does not include information available on social networks or blogs that often contain text written by patients, carers, or relatives.

The team at the HealthNewsReview.org website has proposed a set of 10 criteria to measure the quality of health news that can be applied to stories appearing online or in print (8, 9). This goes beyond the existing HIQ metrics (such as the JAMA score where a website should satisfy four metadata criteria to be considered of good quality, viz. : authorship, source attribution, site ownership disclosure, and currency). While such metrics have been developed primarily to assess online information, the HealthNewsReview.org criteria aim at assessing quality, mainly in terms of completeness of the content, of news stories that include a claim of efficacy about new treatments, devices, dietary recommendation, and other types of health interventions. HealthNewsReview.org publishes assessments of stories according to their criteria, undertaken by a panel of experts. However, manual annotation is time-consuming and cannot be applied to large datasets. An automatic evaluation system could allow the development of tools that help the readers to judge the quality of health news they read online, for instance with a browser add-on.

Machine learning (ML) models were employed previously in the automated quality assessment process. The Health ON the Net (HON) organization, for example, used ML to assess a number of medical websites on whether they comply with a set of eight ethical principles (criteria) defined by HON (10–12). Other work utilized different criteria on different datasets (13, 14). A recent study used the state-of-the-art NLP per-trained deep learning models BERT and BioBERT to automatically annotate of the Brief DISCERN criteria (15). In our previous work (16), we proposed the use of an approach based on natural language processing (NLP) and ML to identify evidence-based advice in health websites as a quality criterion. In this paper, we use an extended methodology to assess the HealthNewsReview.org criteria automatically in news stories, using an annotated dataset of 1,333 news stories obtained from HealthNewsReview.org. We treated the evaluation of the criteria as binary classification tasks, in a manner similar to tasks such as sentiment analysis, sarcasm detection, and argument detection (17–19). A prototype is introduced that detects whether each of the 10 criteria is satisfied. The dataset we used in this study is unique and relatively large compared to other similar studies. We would like to point to the importance of the application area for the health informatics community and we believe the dataset is a novel contribution that other researchers can use.

## 2. Materials and Methods

### 2.1. HealthNewsReview.org

**Figure 1:**
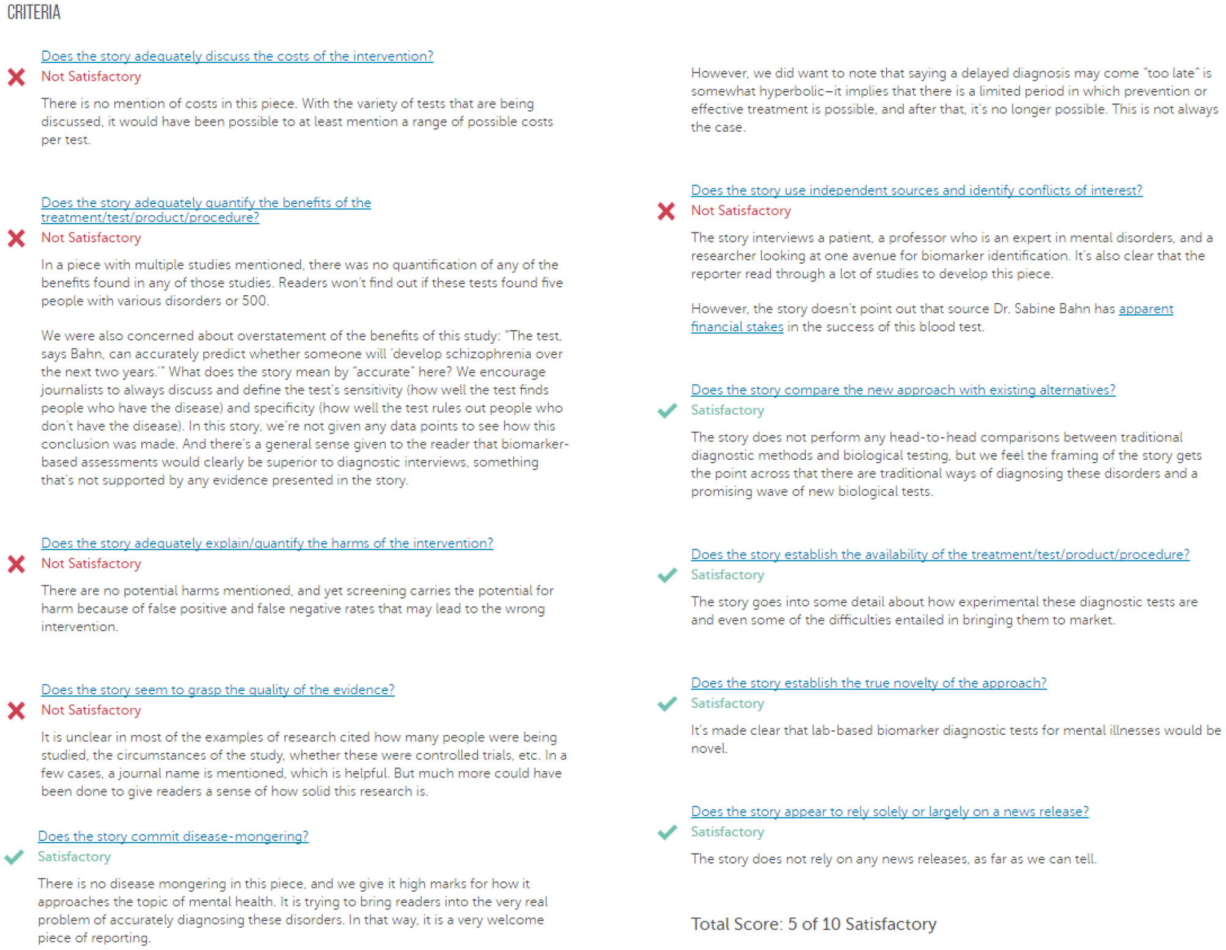
A sample of a review webpage for a news story. (Source: https://www.healthnewsreview.org/review/well-written-newsweeks-look-at-blood-tests-for-mental-illnesses-needed-a-bit-more-hard-data/).

HealthNewsReview.org developed a set of ten criteria, listed in Table 1, to help health consumers analyze the claims of health care interventions critically (8, 9, 20). The review process of scoring the criteria is performed by two or three members of a multidisciplinary team of experienced reviewers, and the 10 criteria are checked for being “Satisfactory,” “Unsatisfactory,” or “Not Applicable.” For each news story, the 10 criteria are scored, and then a total score is calculated and interpreted into a star rating from 0 to 5. In this work we only try to automate scoring the 10 criteria separately without any attempt to calculate the overall score. An example of a review webpage is shown in Figure 1. Notice that in addition to giving the score, the review provides a justification for each assessment. The authors of the website reported that they tested inter-rater reliability before launching the site, using a random sample of 30 stories. Two reviewers coded each story and the average percent agreement between the two reviewers across the ten ratings criteria was 74% (21).

**Table 1:** HealthNewsReview.org Criteria (8).

**No**.	**Criterion**
1	Does the story adequately discuss the costs of the intervention?
2	Does the story adequately quantify the benefits of the treatment/test/product/procedure?
3	Does the story adequately explain/quantify the harms of the intervention?
4	Does the story seem to grasp the quality of the evidence?
5	Does the story commit disease-mongering?
6	Does the story use independent sources and identify conflicts of interest?
7	Does the story compare the new approach with existing alternatives?
8	Does the story establish the availability of the treatment/test/product/procedure?
9	Does the story establish the true novelty of the approach?
10	Does the story appear to rely solely or largely on a news release?

The purpose of the study was to analyze the quality of health news stories, that is news about health published in news outlets aimed at laypersons (magazine, newspapers—online or in print). Of course, there are larger databases of news such as Proquest, Gale (for UK news) and Lexis, and searching health news in these databases would have returned a larger number of samples. However, we needed a dataset of health news stories that had been annotated and graded for criteria of health information quality by health professionals. To our knowledge, the only such dataset is the one available from healthnewsreviews. This dataset has all the news graded for criteria specific for health information quality by a team of reviewers that include medical journalists and medics (20, 22). The size of the dataset is still considerably large for this kind of studies, a recent study posted on arXiv (15) specifically aimed at automating the quality rating on websites on breast cancer, arthritis, and depression was based on a total of 269 documents, much smaller than ours. We would like to note that we intended to use the presented methodology on a larger set of criteria that we proposed for general health information quality assessment (23); however, building a big dataset that is annotated by experts according to these criteria is time-consuming and labor-intensive, so we wanted to utilize a resource of existing annotated data to perform a feasibility study.

#### 2.1.1. The Dataset

Although the website was publicly available (research ethics was not required), not all webpages were easily trackable. Therefore, we had to ask the publisher to get all the review pages' links. After obtaining the links of all the reviews from the HealthNewsReview.org publisher, we extracted the key information for each review. The information includes the link to the review page, the link to the original article, whether the original article online is accessible or not, the value for each criterion (either satisfactory, not satisfactory or not applicable), the number of applicable criteria, the number of “satisfactory” or “not satisfactory” criteria and the total score (the number of satisfactory criteria/the number of applicable criteria). Moreover, the reviews' webpages were saved as HTML files along with the original article webpages (if accessible).

Information about 2,246 reviews of story articles was extracted, of which 913 of the original articles were not accessible (for instance due to a paywall or an obsolete link). The classifications of the 10 criteria for the accessible articles are shown in Table 2. We needed to decide what to do about non-applicable classifications— for “positive” criteria we classified them as unsatisfied, and for “negative” criteria (criteria 5 “disease-mongering” and 10 “relying solely on a news release”) we classified them as satisfied. This is because the latter are negative criteria (the indicators are negatively presented); hence if they were scored “not applicable” in any of the articles they were considered satisfactory. The dataset can be accessed at https://github.com/Maj27/HealthNewsReview/tree/master/Dataset.

**Table 2:** News stories corpus statistics.

**No**.	**Criterion**	**No. of satisfactory**	**No. of unsatisfactory**
		**articles**	**articles**
1	Costs	313 (23.5%)	1020 (76.5%)
2	Benefits	440 (33%)	893 (77%)
3	Harms	470 (35.2%)	863 (64.8%)
4	Quality of the evidence	523 (39.2%)	810 (60.8%)
5	Disease-mongering	1111 (83.3%)	223 (16.7%)
6	Identify conflicts of interest	713 (53.5%)	620 (46.5%)
7	Compare with existing alternative	587 (44%)	746 (56%)
8	Availability of the treatment	864 (64.8%)	469 (35.2%)
9	Novelty of the approach	946 (70.9%)	387 (29.1%)
10	Relying solely on a news release	1265 (94.9%)	69 (5.1%)

### 2.2. The Proposed Model

The aim of this work is to convert the evaluation process so it can be done automatically rather than manually. This is done by identifying the indicators for each of the ten criteria as a classification problem. We used NLP and ML techniques to determine whether the criteria are satisfactory or not by extracting textual features and then training classifiers on those features. First, textual features that indicate the existence of a given criterion are extracted using NLP techniques. After that, we trained ML models on the extracted features to make decisions about whether that criterion is satisfactory or not.

#### 2.2.1. Preprocessing and Setting up the Datasets

For preprocessing, the first step was to set up the corpus to be saved into separate files with the corresponding labels. For that, the saved webpages were automatically cleaned from markup tags and web scripts, as well as other unrelated texts such as advertisements using the beautifulsoup Python library (24). Other text normalizations were also applied, such as removing special characters, lowercasing the text and lemmatization using NLTK WordNetLemmatizer (25). All preprocessing steps were done automatically using Python 2.7 (26).

In order to classify whether articles satisfy the ten criteria or not, we set up ten different datasets. These datasets are all different shufflings (based on each criterion) of the 1,333 documents. For each criterion, two classes were defined using the corresponding labels (“satisfactory” and “not satisfactory”), and the cleaned webpages were split accordingly, and this setting represents one dataset. This resulted in 10 different datasets to be used to train 10 different classifier sets, one for each criterion. These datasets were split into training, validation and testing. For testing, 10% of each dataset documents were saved and set aside to be used as testing sets. For the remaining 90% of the datasets, 80% were used as training sets, and 10% for further validation and hyperparameter-tuning before the final testing.

#### 2.2.2. Features

Textual features are used to expose differences between criteria with satisfactory and non-satisfactory scores. We experimented with different combinations of textual features such as single words, n-grams, word stems and term frequency inverse document frequency (tf-idf). The results obtained using tf-idf feature vectors of lemmatized word n-grams (up to trigrams) were the best, and hence these were used as the features. In addition, we applied other linguistic features. The idea of extending the feature vector to include such features was to reveal how linguistic features could help to identify whether criteria scores are satisfactory or not. Adding domain knowledge information also tends to improve classification accuracy (27). The other reason for utilizing linguistic features was to reduce the high dimensionality of the feature space (also known as Hughes effect), where, as the dimensionality of features increases, the accuracy of a machine learning algorithm decreases (28). Therefore, we tried to reduce the number of features in order to reduce this effect. This is performed by combining related features.

As further linguistic features and to reduce Hughes effect, we used the Named Entity Recognizer tags for each word using the Stanford NER tagger (29). Moreover, we used other linguistic features extracted from Wiktionary inspired and used by Rashkin et al. (30) which they named “intensifying Wiktionary lexicons.” These lexicons comprise five lists of words (viz. comparatives, superlatives, action adverbs, manner adverbs, and modal adverbs) that the authors believe imply a degree a dramatization based on a hypothesis that fake news articles' writers utilize them to attract readers.

Stanford NER was applied to the text before extracting the features, and entities were replaced with the corresponding tags. For example, a mention of an organization is replaced with “ORGANIZATION,” and the same applied to persons' names that were replaced with “PERSON.” Likewise, “DATE,” “LOCATION” tags were added whenever a date or location were encountered. The 7 class model was used in this study with labels (Location, Person, Organization, Money, Percent, Date, and Time) (29). Also, any encountered number was replaced with the word “NUMBER.”

Only the list of comparatives forms out of the five lists of intensifying Wiktionary lexicons used in (30) were used in our experiments. Furthermore, we added three additional lists to them from the same source (https://en.wiktionary.org). These lists are (numerals, auxiliary verbs, degree adverbs). As was done for NER tags, words from these lists appeared in the text were replaced with the corresponding list labels (e.g., “NUMERALS,” “COMPARATIVES,” etc.).

Every task was treated separately and the general features were the same; however, the additional linguistic features were different. For instance, the stories where “compare with other alternatives” criterion is satisfied would probably use comparative forms more often. Hence, for this criterion, the Wiktionary comparative forms were applied. Likewise, this was done for the other datasets where these linguistic features will arguably help to identify whether a criterion score is satisfactory or not. For example, numerals list could help to identify criteria 1–3 (cost, harms, and benefits); while comparatives list could help to identify criteria 2 and 7 (benefits and comparing with other alternatives), etc. Adding these features improved the results, and the performance of the classifiers was better than when experimented without using them.

We did not use, for this purpose, well-known text-mining tools or models such as UMLS (31), Mesh (32), and Biomedical BERT (BioBERT) (33) as these are more fit for text mining the scientific (academic) research literature rather than news stories. The reason is that the text in health news stories is mainly natural and do not contain that many jargons as the news articles target lay people who are not experts in the medical field, unlike the medical literature for which those vocabularies, terminologies, and ontologies have been developed.

#### 2.2.3. Performance Measures and Tools

The performance was analyzed by means of recall, precision and F_1_ measures. Python programming language was utilized to implement the proposed method where Natural Language Toolkit NLTK (25) and the Scikit-learn (34) libraries were mainly used. The code is made available on https://github.com/Maj27/HealthNewsReview.

## 3. The Experiments and Results

To automate the assessment of the ten criteria presented in Table 1, we experimented with five different classifiers. There classifiers are logistic regression (35), support vector machines (SVM) (36), random forests (RF) (37), XGBoost (38), and CatBoost (39). The best performing model on average was CatBoost followed by both SVM and XGBoost. For the sake of a brief presentation in this paper, CatBoost and SVM were selected. The complete list of results of all classifiers is included in Tables A1, A2 in Appendix A. These classifiers were trained and validated using stratified 5-fold cross-validation on the training sets. One of the goals of using cross-validation is to minimize the risk of overfitting. However, we observed that overfitting was occurring with larger numbers of features, even with cross-validation, making effective feature selection difficult. To overcome this, we held out a second subset of the data as a validation set, which we could use to monitor overfitting of the cross-validation training during the feature selection process. Feature selection was then a trade-off between adding more features to improve performance on the training data, but not so many that performance on the validation data dropped (due to overfitting).

For each task, we experimented with different feature vector lengths to see how many features are needed to be considered by the classifiers in order to produce the best performance. This was done using the Python SelectKBest which is a univariate feature selection that works by selecting the best features based on univariate statistical tests. The following cutoffs were tried in the experiments to select the top n features [50, 200, 500, 1,000]. As we treat these tasks separately, this was done to all 10 tasks and different numbers of features were selected for each of them. The cuts were decided by taking the minimum number of features that yielded the best results on the training data and varied between the tasks and across the different classifiers. For some, a small number of features was enough, such as in criterion 2 (benefits) where 100 were enough for SVM to give the best performance. Whereas for the others, more features were required, such as criterion 7 (compare with existing alternatives) where the cut was 500. It is worth mentioning that the cuts were higher before incorporating the linguistic features (NER and Wiktionary lists).

**Figure 2:**
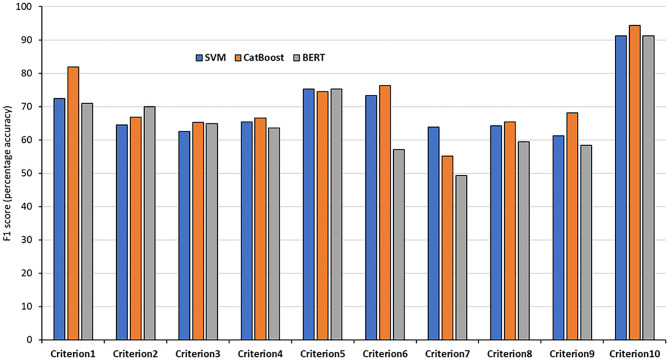
F_1_ results of the 10 testing sets. The results of each criteria using the SVM, CatBoost, and BERT classifiers.

In order to achieve the best performance using the mentioned classifiers, we used gridsearch to search for sets of hyperparameters that result in the best performance of the models on each task. This is known as hyperparameter tuning (Table 3). After tuning the classifiers' parameters and using different thresholds for feature generation and selection, we obtained relatively good classifications figures on the training and validation sets, as shown in Table 4. The system was then evaluated on the testing sets with the same setup. The results of the testing sets are shown in Table 5 and summarized in Figure 2. Additionally, we experimented with the use of pre-trained natural language model BERT (40) to compare the results of our method based on feature engineering and the state of the art NLP techniques. The results obtained using BERT are also shown in Table 3 for comparison.

**Table 3:** Employed models with the corresponding hyperparameters.

**Classifier**	**Hyperparameters**
LR	C: [0.1, 1.0]
	penalty: [ 'l2', None],
	solver: [ 'lbfgs', 'liblinear'],
	max_iter:[100, 200],
	class_weight:[None, 'balanced']
SVM	kernel: ['rbf','linear'],
	C': [1, 8,10]
RF	max_depth: [5, 10, 30, None],
	criterion:['gini','entropy'],
	bootstrap: [True],
	max_features:['log2', None],
	n_estimators: [50, 100, 200, 400]
XGB	learning_rate: [0.05, 0.1, 0.15],
	bytree:[.5, 1],
	max_depth: np.arange(3, 6, 10),
	n_estimators: [50, 100, 200, 400]
CB	learning_rate: [0.05, 0.1, 0.15]

**Table 4:** Results of the ten validation sets using SVM and CatBoost.

**No**.	**Criterion**	**Classifiers**	**Recall**	**Precision**	**F_1_**
1	Costs	SVM	79.7	80.41	73.95
		CatBoost	79.7	77.6	77.61
2	Benefits	SVM	69.4	67.78	68.1
		CatBoost	71.64	70.07	69.98
3	Harms	SVM	76.69	78.34	73.5
		CatBoost	76.69	76.57	74.61
4	Quality of the evidence	SVM	64.66	63.3	62.22
		CatBoost	64.66	63.3	62.22
5	Disease-mongering	SVM	83.46	69.65	75.93
		CatBoost	84.21	86.72	77.68
6	Identify conflicts of interest	SVM	71.43	72.02	70.89
		CatBoost	67.67	67.6	67.54
7	Compare with existing alternative	SVM	53.03	52.34	52.5
		CatBoost	50.76	49.9	50.1
8	Availability of the treatment	SVM	65.41	62.52	61.6
		CatBoost	66.92	64.77	64.17
9	Novelty of the approach	SVM	71.21	65.58	61.76
		CatBoost	73.48	70.98	68.54
10	Relying on a news release	SVM	95.45	91.12	93.23
		CatBoost	96.97	97.06	96.24

**Table 5:** Results of the ten testing sets using SVM, CatBoost, and BERT.

**No**.	**Criterion**	**Classifiers**	**Recall**	**Precision**	**F_1_**
1	Costs	SVM	79.26	83.69	72.43
		CatBoost	83.7	82.99	81.94
		BERT	74.07	69.89	70.99
2	Benefits	SVM	70.15	68.68	64.56
		CatBoost	70.15	67.98	66.8
		BERT	71.64	70.07	69.98
3	Harms	SVM	64.93	62.49	62.61
		CatBoost	69.4	68.36	65.29
		BERT	64.93	64.76	64.84
4	Quality of the evidence	SVM	67.41	66.67	65.39
		CatBoost	68.15	67.42	66.6
		BERT	64.44	63.51	63.61
5	Disease-mongering	SVM	82.96	68.83	75.24
		CatBoost	81.48	68.62	74.5
		BERT	82.96	68.83	75.24
6	Identify conflicts of interest	SVM	73.33	73.33	73.33
		CatBoost	76.3	76.27	76.27
		BERT	58.52	58.44	57.13
7	Compare with existing alternative	SVM	64.71	64.45	63.86
		CatBoost	58.09	57.16	55.17
		BERT	49.26	49.35	49.31
8	Availability of the treatment	SVM	68.42	66.48	64.25
		CatBoost	67.67	65.63	65.44
		BERT	66.17	62.78	59.4
9	Novelty of the approach	SVM	71.32	70.03	61.3
		CatBoost	73.53	71.98	68.09
		BERT	70.59	49.83	58.42
10	Relying on a news release	SVM	94.12	88.58	91.27
		CatBoost	95.59	95.79	94.32
		BERT	94.12	88.58	91.27

The classification accuracies for most of the criteria on both the validation and testing sets were good and even better than the training accuracies. For some criteria the results were promising. The F_1_ scores for criterion 1 (cost), for example, were 77.61% and 81.94% using the CatBoost classifier in both validation and testing sets, respectively. The F_1_ scores of criteria 2, 3, 5, and 6 (benefits, harms, disease-mongering, and identify conflicts of interest) were also good. The results of the other tasks were average, justifications are discussed in the discussion section. On the other hand, other tasks, especially criterion 7 (comparing with other alternatives), seem harder to classify. This is discussed in the next section. In general, traditional models outperformed BERT in the presented tasks. We argue that this is due to the power of handcrafting and selecting the most informative features for those tasks. Additionally, we think the main reason is that the dataset is not big enough to apply deep learning methods. Hence, the performance degraded when using BERT. We believe if the dataset was bigger, pre-trained models would have performed differently.

We also examined the 100 highest-weighted features used by the SVM classifier for each task. We found that for criterion 2 (benefits) for instance, features such as “AUXILIARY_VERB increased,” “AUXILIARY_VERB improve,” “AUXILIARY_VERB effective,” and “AUXILIARY_VERB also help” were amongst the top features that the classifier relied on when classifying the documents. For criterion 3 (harms), side effects phrases and words such as “DEGREE_ADVERB major,” “DEGREE_ADVERB common,” “cancer,” “effective,” were the prominent features. The same goes for cost where words and phrases that contain numbers, percentages and cost phrases were at the top, etc. It is also worth mentioning that the linguistic aggregated features appear always at the top ranks of the features and that is why we believe using the handcrafted features gave the classifiers the ability to perform well and produce better results than those obtained by BERT were no such preprocessing is done.

## 4. Discussion

The proposed method showed that it is feasible to use classification as a way of scoring the quality criteria in news stories, although the performances vary among criteria. For some criteria, such as the mention of costs and “disease-mongering” (criteria 1 and 5) followed by mention of harms and benefits (criteria 3 and 2), the tasks were relatively easier to automate. However, for other tasks such as comparing with other alternatives and availability of treatment (criteria 7 and 8) the performance was relatively poor. High performance judging criteria 10 was also observed; however, we cannot rely on these results as the distribution of the two classes is imbalanced [the number of documents belonging to the positive class is significantly higher (94.9%) than those belonging to the negative class].

Nevertheless, there were still samples that were misclassified. We believe that these samples were difficult and more ambiguous for the classifiers to identify. Table 6 shows some of these samples with some justifications. In order to understand why the performance varied between different criteria, we considered the following possible explanations:

**Table 6:** Examples of misclassified documents by classifiers.

**No**.	**Criterion**	**False type**	**File**	**Comments**
1	Costs	FP	Article_1727	The cost of the treatment is mentioned ($2,400) and that is why it was picked by the classifiers. However, the reviewers attributed this to the incomplete assessment of costs and other side effects might occur in the future with no consideration of their treatment costs.
2	Benefits	FP	Article_68	The article does not quantify benefit, just writes led in decrease in insulin levels. Maybe the classifier was misled by an earlier sentence describing the intervention with words often used to describe the efficacy (participants reduced calories by 25%, but this is the inter-vention, not the result)
3	Harms	FN	Article_1034	The article mentions that it does no harm, but in such a generic way Exhibiting few side effects; and this would be difficult for the classifier as phrases such as side effects would confuse the classifier.
4	Quality of the evidence	FN	Article_1050	The story mentions evidence without being very specific (has under-gone several successful initial trials including one lasting 1 year. However it honestly says a larger study has just began it will take a year or more to assemble data.
5	Disease-mongering	FP	Article_739	This story is about diet, not standard treatment and probably this is a difficult epistemological problem.
6	Identify con-flicts of interest	FP	Article_735	The story mentions finding by a consortium, however, the reviewers were strict and asked for additional source with deep knowledge in the field
7	Compare with existing alterna-tive	FP	Article_941	Here the intervention is circumcision and this maybe very different from usual treatments.
8	Availability of the treatment	FN	Article_1020	It is mentioned in the story that they filed a patent so it is implicit it may take time.
9	Novelty of the approach	FP	Article_542	This seems to be about side effects of Botox not the classical news on new treatment/diagnostics/nutritional advice.

**The small size of the dataset**

Although the number of samples used was around 1,300, this sample size is not sufficient for many NLP classification problems. Usually, in NLP-based ML algorithms, the more data is used to train the model, the more accurate and generalizable the model output will be (41). This can be inferred from the different sizes and results of most presented criteria when moving from training sets, validation and testing sets where as the training data increases in this order, the performance improves.

**The variability of the writing styles**

As the articles used came from various sources that were written by different people, it would be plausible that these articles would be of different writing styles. Hence there is a difficulty due to the high representation variations in the textual information provided by various writers. Increasing the size of the dataset might help to solve this problem. Studies such as (42) pointed to the affect of different writing styles in classification tasks.

**The need of external knowledge**

Scoring some of the criteria sometimes needs external knowledge. Whether a story relies on news release (criterion 10), for instance, is an example of requiring external knowledge as sometimes it might not be enough to judge from the text only, and one needs to check other news releases in the same period of time (typically, if a news article is arising from a press release, a similar story will be published by several news outlets). One could think of searching press releases on press release websites such as eurekalert.org, medicalxpress.com, sciencealert.com). Also, in order to compare the new intervention with other alternatives (criterion 7), one needs to be aware of such alternatives, and this usually requires external knowledge, for instance of the clinical guidelines. The National Institute Of Clinical Excellence (NICE) guidelines https://www.nice.org.uk/guidance is a source that one might look up for such guidelines. This makes judging this criterion quite challenging without such knowledge. Therefore, it is unlikely that NLP can replace expert judgment by a panel of experts. Automating the process of finding and integrating external knowledge is a challenging problem as they are not available in accessible databases. There is research that studies automatic knowledge extraction for acquiring disease-specific knowledge (43).

**The subjectivity in the manual scoring of some of the criteria**

When judging the satisfaction of each criterion, different evaluators have different perspectives about whether a criterion is satisfied or not, especially over an extended period of time. Although scoring is made by a panel, which reduces subjectivity, there may still be variations in the assessment for some of the criteria. For example, disease-mongering criterion (criterion 5) is highly subjective, and it is a matter of judgment. According to the publisher of the website, it is the most difficult criterion for the reviewers to apply (44); and often there is a fine line about whether an article on some diseases that might be serious for some sufferers is misrepresenting the condition to the public. For another example, when a criterion is not applicable, it is considered by some evaluators as passing the satisfaction criterion, although very rarely, because it was deemed not needed. Such a case confuses the classifiers as there is no indication (feature coefficients) of the existence of that criterion.

**The confusion of partial criteria indicators**

For other criteria, such as the “benefits” criterion (criterion 2), mentioning partial benefits for an intervention is sometimes not adequate to satisfy this criterion. Some articles, for instance, may mention the benefits of the intervention but the evaluators would consider this unsatisfactory if it does not comprehensively mention all the benefits in all cases. The same applies for mentioning the side effects (criterion 3), where evaluators may consider mentioning only some potential adverse effects as insufficient. Moreover, some evaluators go beyond that, and question whether the improvement being statistically significant means that is it clinically significant? As a result, the existence of these textual indicators (although partially satisfied) are interpreted by the classifiers as authentic features that eventually affect the results. Table 6 shows some examples of difficult cases where the classifiers performed poorly.

A major limitation of the models presented in the study is that they only apply to news articles about health and cannot be generalized because the information quality criteria used are specific to health news stories. In fact, other types of communication, for instance, social media or forums, use different styles of language and it is likely that a different methodology, and different means of grading of health information quality, will be needed. Although this is a major limitation, this data set is rich and unique in terms of the genre especially there is a lack of similar datasets in the literature that researchers can use in such studies.

## 5. Conclusion and Future Directions

In this paper, we proposed a method to automatically evaluate the quality criteria of health news stories developed by the HealthNewsReview.org. These criteria are more challenging than those addressed by previous work (10, 16), especially when compared to metadata criteria concerning checking the timeline of the articles or revealing the authors of the content, etc. Our aim was to investigate how much more difficult the machine learning task was, and how and why it varied between criteria. We explored features ranging from simple n-grams to more advanced linguistic features and optimized the feature selection for each task. For some criteria, the results obtained were promising; however, automated evaluation of the other criteria may not yet replace the manual evaluation process where human experts interpret text senses and make use of external knowledge in their assessment. The results are limited to judge the appropriateness of the automation of this evaluation method based on the ten studied criteria, and not to evaluate health news in general.

Our future work will explore two complementary directions. On the one hand, we plan to go beyond the multiple binary classifications and try using deep learning to score the 10 criteria as a multi-class, multi-label classification problem (45), which may reveal and exploit any correlation between the different tasks. On the other hand, we will explore whether there are simpler objective versions of these criteria which retain the utility of the distinction being made here but are more amenable to automatic evaluation. Additionally, there are many other criteria in the literature that their applicability for the automatic assessment can be studied (23). This method can be extended and applied to such criteria to judge its appropriateness. In this study, we were limited to the presented 10 criteria. We believe this methodology can be made generalizable to other datasets that use different criteria should suitable annotated datasets are available.

## Data Availability Statement

All datasets generated for this study are included in the article/Supplementary Material.

## Author Contributions

MA-J designed the research study, conducted all experiments and analyses, and wrote the manuscript. RE supervised the study and reviewed the manuscript. JL reviewed and revised the manuscript. PG rewrote the discussion section, revised the manuscript and contributed in the analysis.

## Conflict of Interest

The authors declare that the research was conducted in the absence of any commercial or financial relationships that could be construed as a potential conflict of interest.
