# Supplementary material for: Automatic Identification of Information Quality Metrics in Health News Stories
Source: Front Public Health. 2020 Dec 18;8:515347. doi: 10.3389/fpubh.2020.515347 (PMC7775604; doi:10.3389/fpubh.2020.515347)
Supplement: Supplementary file 1 [file Data_Sheet_1.PDF]

APPENDIX A

Table A1: Validation set Results

Criteria	Classifier	Recall	Precision	F1
Criterion1				
	Logistic Regression	76.69	58.82	66.57
	SVM Classifier	79.7	80.41	73.95
	RandomForest Classifier	76.69	70.78	67.9
	XGBoost Classifier	78.95	76.52	76.55
	CatBoost Classifier	79.7	77.6	77.61
Criterion2				
	Logistic Regression	66.92	44.78	53.65
	SVM Classifier	76.69	78.34	73.5
	RandomForest Classifier	69.92	67.93	65.54
	XGBoost Classifier	68.42	65.81	65.37
	CatBoost Classifier	76.69	76.57	74.61
Criterion3				
	Logistic Regression	64.66	41.81	50.78
	SVM Classifier	78.2	78.49	76.77
	RandomForest Classifier	73.68	77.34	69.22
	XGBoost Classifier	74.44	73.72	73.4
	CatBoost Classifier	74.44	74.09	72.62
Criterion4				
	Logistic Regression	61.65	76.47	47.8
	SVM Classifier	64.66	63.3	62.22
	RandomForest Classifier	64.66	63.3	62.22
	XGBoost Classifier	67.67	66.83	66.05
	CatBoost Classifier	64.66	63.3	62.22
Criterion5				
	Logistic Regression	83.46	69.65	75.93
	SVM Classifier	83.46	69.65	75.93
	RandomForest Classifier	83.46	69.65	75.93
	XGBoost Classifier	84.21	86.72	77.68
	CatBoost Classifier	84.21	86.72	77.68
Criterion6				
	Logistic Regression	65.41	68.26	62.91
	SVM Classifier	71.43	72.02	70.89
	RandomForest Classifier	69.17	69.17	68.97
	XGBoost Classifier	72.93	74.27	72.16
	CatBoost Classifier	67.67	67.6	67.54
Criterion7				
	Logistic Regression	54.55	51	47.42
	SVM Classifier	53.03	52.34	52.5
	RandomForest Classifier	50.76	49.48	49.64
	XGBoost Classifier	50	48.31	48.44
	CatBoost Classifier	50.76	49.9	50.1

Criterion8				
	Logistic Regression	65.41	77.47	52.49
	SVM Classifier	65.41	62.52	61.6
	RandomForest Classifier	60.9	54.99	55.35
	XGBoost Classifier	61.65	57.86	58.22
	CatBoost Classifier	66.92	64.77	64.17
Criterion9				
	Logistic Regression	71.21	50.71	59.24
	SVM Classifier	71.21	65.58	61.76
	RandomForest Classifier	68.94	61.67	62.33
	XGBoost Classifier	72.73	69.61	67.29
	CatBoost Classifier	73.48	70.98	68.54
Criterion10				
	Logistic Regression	95.45	91.12	93.23
	SVM Classifier	95.45	91.12	93.23
	RandomForest Classifier	95.45	91.12	93.23
	XGBoost Classifier	96.21	96.36	94.9
	CatBoost Classifier	96.97	97.06	96.24

Table A2: Test set Results

Criteria	Classifier	Recall	Precision	F1
Criterion1				
	Logistic Regression	76.3	58.21	66.04
	SVM Classifier	79.26	83.69	72.43
	RandomForest Classifier	77.78	82.79	69.39
	XGBoost Classifier	76.3	73.15	73.73
	CatBoost Classifier	83.7	82.99	81.94
	BERT	74.07	69.89	70.99
Criterion2				
	Logistic Regression	67.16	45.11	53.97
	SVM Classifier	70.15	68.68	64.56
	RandomForest Classifier	68.66	65.95	62.07
	XGBoost Classifier	70.15	68.12	65.77
	CatBoost Classifier	70.15	67.98	66.8
	BERT	71.64	70.07	69.98
Criterion3				
	Logistic Regression	64.93	42.15	51.12
	SVM Classifier	64.93	62.49	62.61
	RandomForest Classifier	65.67	62.6	57.1
	XGBoost Classifier	67.16	65.25	65.18
	CatBoost Classifier	69.4	68.36	65.29
	BERT	64.93	64.76	64.84
Criterion4				
	Logistic Regression	60.74	36.89	45.91
	SVM Classifier	67.41	66.67	65.39
	RandomForest Classifier	68.89	69.25	65.96
	XGBoost Classifier	70.37	70.05	68.81
	CatBoost Classifier	68.15	67.42	66.6
	BERT	64.44	63.51	63.61
Criterion5				
	Logistic Regression	82.96	68.83	75.24
	SVM Classifier	82.96	68.83	75.24
	RandomForest Classifier	82.96	68.83	75.24
	XGBoost Classifier	80	68.4	73.74
	CatBoost Classifier	81.48	68.62	74.5
	BERT	82.96	68.83	75.24
Criterion6				
	Logistic Regression	71.85	73.04	71.07
	SVM Classifier	73.33	73.33	73.33
	RandomForest Classifier	77.04	77.02	77.02
	XGBoost Classifier	70.37	70.33	70.33
	CatBoost Classifier	76.3	76.27	76.27
	BERT	58.52	58.44	57.13
Criterion7				

	Logistic Regression	55.88	31.23	40.07
	SVM Classifier	64.71	64.45	63.86
	RandomForest Classifier	58.82	58.07	56.14
	XGBoost Classifier	58.09	57.3	56.98
	CatBoost Classifier	58.09	57.16	55.17
	BERT	49.26	49.35	49.31
Criterion8				
	Logistic Regression	65.41	42.79	51.74
	SVM Classifier	68.42	66.48	64.25
	RandomForest Classifier	63.91	59.17	58.52
	XGBoost Classifier	66.17	63.41	62.98
	CatBoost Classifier	67.67	65.63	65.44
	BERT	66.17	62.78	59.4
Criterion9				
	Logistic Regression	71.32	79.61	60.11
	SVM Classifier	71.32	70.03	61.3
	RandomForest Classifier	71.32	79.61	60.11
	XGBoost Classifier	72.79	74.72	64.31
	CatBoost Classifier	73.53	71.98	68.09
	BERT	70.59	49.83	58.42
Criterion10				
	Logistic Regression	94.12	88.58	91.27
	SVM Classifier	94.12	88.58	91.27
	RandomForest Classifier	94.12	88.58	91.27
	XGBoost Classifier	94.12	88.58	91.27
	CatBoost Classifier	95.59	95.79	94.32
	BERT	94.12	88.58	91.27
